# Editorial: Serine protease inhibitors and their therapeutic applications

**DOI:** 10.3389/fchem.2022.980152

**Published:** 2022-08-08

**Authors:** Letizia Crocetti, Mark T. Quinn, Agostino Cilibrizzi, Maria Paola Giovannoni

**Affiliations:** ^1^ NEUROFARBA, Pharmaceutical and Nutraceutical Section, University of Florence, Sesto Fiorentino, Italy; ^2^ Department of Microbiology and Cell Biology, Montana State University, Bozeman, MT, United States; ^3^ Institute of Pharmaceutical Science, King’s College London, London, United Kingdom

**Keywords:** serine proteases, inhibitors, drug design, organic synthesis, molecular modeling (MD)

Serine proteases are important signaling molecules and their dysregulation has been widely associated with pathologies ranging from cardiovascular and inflammatory disorders to cancer and neurological diseases. Undoubtedly, this class of enzymes represents an attractive target for bioactive molecules able to modulate their activity, thus contributing to the resolution of the above-mentioned pathologies. Although many peptides/peptidomimetics, natural products and small-organic molecules have been reported in the literature as potent serine protease inhibitors, the identification of new selective inhibitors, as well as the pharmacological validation of established hits, are still a challenge in medicinal chemistry. This special issue does not aim to be comprehensive, but rather to highlight some increasingly important aspects of serine protease-associated diseases, as well as recent advances in the field.

Burster et al. provide an overview of the physiopathology of neutrophil-derived serine proteases in the development of inflammatory disease. They provide a nice coverage of the role of neutrophil serine proteases in the cardiovascular and respiratory systems, where they have been shown to play roles in regulating thrombosis and in the tissue, damage associated with chronic obstructive pulmonary disease (COPD) and acute respiratory distress syndrome (ARDS). The authors also provide a summary of the role of neutrophil serine proteases in inflammation, with an emphasis on cathepsin G (CatG), neutrophil elastase (NE), and proteinase 3 (PR3). There is also an interesting discussion of the coordination of CatG and lactoferrin and the ability of lactoferrin to augment CatG activity. There is an extensive discussion of serine neutrophil protease inhibitors as therapeutics for the management of a number of diseases where the imbalance between protease activity and endogenous inhibitors can lead to chronic disorders. The authors also suggest a possible correlation between the potent activity of neutrophil serine proteases, especially NE and CatG, and the severe complications observed in COVID-19, thus introducing a new application for serine proteases inhibitors (Zerimech et al., 2021). Indeed, clinical trials with several serine protease inhibitors are being pursued in SARS-CoV-2 management, although the results have been mixed. Nevertheless, it is clear form this review that unregulated neutrophil serine protease activity can be associated with a number of inflammatory diseases, including SARS-CoV-2, and that the development of novel targeted serine protease inhibitors to address this issue is an area of interest.

The contribution from Zolotov et al. reports a study on the synthesis and characterization of five novel cyanopyrrolidine-based compounds designed as analogues of KYP-2047, a potent propyl oligopeptidase (POP) inhibitor. POP is a cytosolic serine peptidase widely distributed in the central nervous system and is involved in several neurodegenerative pathologies, including Parkinson’s and Alzheimer’s diseases (Svarcbahs et al., 2019). Within the new library of compounds developed by Zolotov et al., CbzMetPrdN and CbzGlnPrdN show an inhibitory activity which is comparable to the reference KYP-2047 (i.e. IC_50_ ∼ 2 nM). Moreover, these new molecular hits, demonstrate beneficial anti-amnesic effects in an *in vivo* model of scopolamine and MES-induced amnesia, suggesting the possibility to use POP inhibitors for the treatment of dementia associated to Alzheimer’s disease. The BBB permeability predicted via three molecular descriptors for all the novel POP inhibitors reported in the study has been also confirmed *in vivo* for CbzMetPrdN, which results able to inhibit POP activity when administered intraperitoneally. Lastly, molecular docking studies into POP active site highlight that this new series of compounds adopt an orientation in the POP-binding site which overlaps with the pose of the reference compound KYP-2047.

The paper of Ferguson et al. reports a series of peptide-based irreversible inhibitors of trypsin and trypsin-like proteases (TLPs) obtained using solid-phase peptide synthesis. The new molecules contain an N-alkyl glycine analogue of arginine, and bear different electrophilic leaving groups, such as carbamate and triazole urea. The evaluation of their selectivity against neutrophil elastase and chymotrypsin has been performed by the authors and NAP858 results to be the most potent and selective inhibitor with a Ki = 0.2 µM (Chen et al., 2019). NAP858 and other compounds have been further evaluated as activity probes (APs) for trypsin and for a range of different TLPs, highlighting potential benefits for the detection and purification of proteases in extremely complex biological samples. Moreover, due to the straightforward synthetic process the peptide-based inhibitors described in this study could be useful in to setup Activity-Based Profiling applications, enabling the rapid screening of new trypsin and TLP inhibitors.

Finally, Ceuleers and coworkers contribute with a manuscript concerning the role of serine proteases on visceral pain in different rodent models characterized by an intestinal insult. In this regard, serine proteases are known to play a key role in the origin of abdominal pain in inflammatory bowel diseases (IBD, such as Chron’s disease) and irritable bowel syndrome (IBS) (Ceuleers et al.). Ceuleers et al. (2018) further investigated the effects of serine protease inhibitors Nafamost mesylate and UAMC-00050 on visceral hypersensitivity, using two different mouse models - i.e. TNBS-acute colitis for Chron’s disease and chronic neonatal acetic acid mouse model for IBS. The outcome of this study highlights that Nafamost mesylate and UAMC-00050are more effective in reducing visceral allodynia and hyperalgesia in the chronic model of colitis, rather than in the acute model. In addition, a serine protease profiling was carried in the colonic wall of acute colitis rats, focusing on the quantification of the mRNA expression of a panel of serine proteases and mast cell tryptase by immunohistochemistry. The results, sometimes in contrast to previous studies, nevertheless provide an interesting perspective for individualized treatments for patients with IBD and IBS.

Our aim in editing this special issue was to expand the range of original papers in the broad field of serine protease targeting and its pharmacological relevance. The collection gives access to a useful set of up-to-date manuscripts, highlighting, interesting aspects and results in the field of serine proteases inhibitors and their possible therapeutic applications, for instance in the treatment of abdominal pain in gastrointestinal diseases, severe complications in COVID-19 and dementia in Alzheimer’s disease.
